# 意义未明的单克隆免疫球蛋白血症和冒烟型多发性骨髓瘤全程管理中国专家共识（2025年版）

**DOI:** 10.3760/cma.j.cn121090-20241122-00469

**Published:** 2025-03

**Authors:** 中华医学会血液学分会浆细胞疾病学组 中国医师协会多发性骨髓瘤专业委员会

## Abstract

意义未明的单克隆免疫球蛋白血症（monoclonal gammopathy of undetermined significance, MGUS）是浆细胞疾病中最常见的一种类型。特征为骨髓内浆细胞克隆性增生、单克隆免疫球蛋白（monoclonal protein, M蛋白）轻度升高，无器官功能损害。冒烟型多发性骨髓瘤（smoldering multiple myeloma, SMM）是介于MGUS和活动性多发性骨髓瘤（active multiple myeloma, AMM）之间的中间疾病阶段的浆细胞疾病，其特征是血浆中M蛋白水平升高，骨髓中浆细胞浸润增加，但尚未出现典型临床表现。SMM被认为是AMM的前驱状态。本共识由中华医学会血液学分会浆细胞疾病学组和中国医师协会多发性骨髓瘤专业委员会共同制定，内容涵盖了MGUS和SMM的流行病学特征、临床表现、检验和检查、诊断标准、鉴别诊断、预后评估以及患者管理策略。本共识旨在为MGUS和SMM的全程管理提供标准化指导，确保及时监控疾病进展，并在适当时期采取干预措施，以提升患者的生活质量和生存率。

## Abstract (en)

Monoclonal gammopathy of undetermined significance（MGUS）is the most common type of plasma cell disorder, characterized by clonal proliferation of plasma cells in the bone marrow, a mild increase in monoclonal protein（M protein）, and no organ damage. Smoldering multiple myeloma（SMM）is a plasma cell disease that lies between MGUS and active multiple myeloma（AMM）, featuring elevated levels of M protein in the plasma and increased plasma cell infiltration in the bone marrow, but without typical clinical manifestations. SMM is considered as a precursor state to AMM. This consensus was jointly developed by the Plasma Cell Disease Group, Chinese Society of Hematology, Chinese Medical Association and the Chinese Myeloma Committee-Chinese Hematology Association, covering the epidemiological characteristics, clinical manifestations, testing and examination, diagnostic criteria, differential diagnosis, prognosis assessment, and patient management strategies for MGUS and SMM. The consensus aims to provide standardized guidance for the comprehensive management of MGUS and SMM, ensuring timely monitoring of disease progression and intervention at the appropriate time to improve the quality of life and survival rates of patients.

意义未明的单克隆免疫球蛋白血症（monoclonal gammopathy of undetermined significance, MGUS）是浆细胞疾病中最常见的一种类型，以骨髓内浆细胞克隆性增生、单克隆免疫球蛋白（monoclonal protein，M蛋白）轻度升高、没有器官功能损害为特征。MGUS转归不一，有些患者可以长期稳定，有些患者可进展为各种恶性浆细胞/B淋巴细胞疾病，如多发性骨髓瘤（multiple myeloma，MM）、淋巴浆细胞淋巴瘤（lymphoplasmacytic lymphoma，LPL）/华氏巨球蛋白血症（Waldenström's macroglobulinemia，WM）、系统性轻链型淀粉样变性（light chain amyloidosis，AL）等。

冒烟型MM（smoldering multiple myeloma，SMM）是介于MGUS和活动性MM（active multiple myeloma，AMM）之间的中间疾病阶段的浆细胞疾病，其特征是血浆中M蛋白水平升高，骨髓中浆细胞浸润增加，但尚未出现典型的临床表现。SMM是AMM的前驱状态，SMM进展到AMM的中位时间约为5年。

目前我国缺乏MGUS和SMM诊治的专家共识。中华医学会血液学分会浆细胞疾病学组和中国医师协会多发性骨髓瘤专业委员会组织37名分布于全国大部分省份的该领域专家，结合国内外MGUS和SMM最新指南和研究进展，编写此共识，旨在为MGUS和SMM的全程管理提供标准化指导，确保及时监控疾病进展，并在适当时机采取干预措施，以提升患者的生活质量和生存率。

一、MGUS

1. 流行病学：不同国家MGUS的患病率存在差异（0.7％～5.8％），其发病率与性别、种族和年龄有关，男性发病率高于女性，黑种人的发病率是白种人的2～3倍，患病率随年龄增长而增加[1]。

一项来自北京协和医院的基于154 597名体检人群的研究发现，MGUS的患病率为0.53％（843名），中位年龄为58岁。在50岁及以上人群中，MGUS的总体患病率为1.11％；在70岁及以上人群中，患病率为2.57％；男性的患病率高于女性（0.66％对0.39％，*P*<0.001）[2]。另一项我国多中心前瞻性研究招募了1 797名健康受试者，MGUS的总体患病率为2.73％，随着年龄增加，患病率逐渐增高，其中41～50岁组1.19％，51～60岁组1.16％，61～70岁组2.19％，71～80岁组3.66％，81岁及以上组7.76％[3]。一项在冰岛进行的基于大规模人群的筛查研究（iStopMM研究）筛查了冰岛所有40岁以上的成年人，包括75 422名受试者，MGUS的发生率为4.4％[4]–[5]。

2. 临床表现：基于2014国际骨髓瘤工作组（International Myeloma Working Group，IMWG）[6]和《中国多发性骨髓瘤诊治指南（2024年修订）》[7]中关于MGUS的诊断标准，MGUS除了发现M蛋白和克隆性浆细胞外，没有浆细胞异常增殖引起的终末器官功能损害，如骨髓瘤引起的相关表现，包括靶器官损害表现（CRAB）、出现1项或多项指标异常（SLiM）（后续统称为SLiM-CARB），以及肝脾淋巴结肿大；也没有其他M蛋白直接或间接引起的器官损害，如AL、M蛋白相关脏器损害（肾脏、心脏、血管、神经、眼部、肌肉、皮肤等）。若临床出现上述症状，必须排除与M蛋白相关才能诊断MGUS。

其中骨髓瘤引起的相关表现详见《中国多发性骨髓瘤诊治指南（2024年修订）》[7]。

CRAB：

［C］校正血清钙>2.75 mmol/L；

［R］肾功能损害（肌酐清除率<40 ml/min或血清肌酐>177 µmol/L）；

［A］贫血（HGB较正常下限降低≥20 g/L或<100 g/L）；

［B］溶骨性破坏，影像学检查（X线、CT或PET‑CT）显示1处或多处溶骨性病变。

SLiM：

［S］骨髓单克隆浆细胞比例≥60％；

［Li］受累/非受累血清游离轻链（serum free light chain，sFLC）比值≥100（受累轻链数值至少≥100 mg/L）；

［M］MRI检测有>1处5 mm以上的局灶性骨质破坏。

3. 检验和检查：

（1）血液/尿液检查：在诊断时，因需要与其他M蛋白相关疾病［如MM、LPL/WM或其他恶性疾病、AL、POEMS综合征、有肾脏意义的单克隆免疫球蛋白血症（monoclonal gammopathy of renal significance，MGRS）、IgM相关性周围神经病变等］鉴别，应接受血常规、血清肌酐（估算肾小球滤过率）、血清钙（计算校正钙）、血清乳酸脱氢酶、β_2_微球蛋白、血清白蛋白、血清免疫球蛋白定量、sFLC、血清蛋白电泳、血清免疫固定电泳、尿微量蛋白定量、24 h尿蛋白定量、24 h尿轻链定量、尿免疫固定电泳检测。

（2）骨髓细胞形态学、流式细胞术及遗传学检查：关于有M蛋白的患者是否需要完善骨髓检查有一定争议。欧洲骨髓瘤小组推荐，对于无症状且血清M蛋白水平≤15 g/L的IgG型MGUS患者可不进行骨髓检查，除非有证据显示疾病进展到有症状阶段。对于所有IgA型和IgM型M蛋白患者，应常规进行骨髓检查[8]。英国血液病学会提出，可以根据MGUS的危险度分层决定是否需要行骨髓穿刺检查[9]。上述共识的提出基于一些回顾性研究数据，数据显示，M蛋白浓度<15 g/L的患者患MM的概率非常低。意大利帕维亚大学Mangiacavalli等[10]报道的一项单中心大宗回顾性研究纳入1 217例患者，表明在没有骨痛症状且M蛋白浓度<15 g/L或10 g/L的患者中，骨髓浆细胞浸润率达到10％的比例非常低，分别为7.3％和5.0％，这种风险与免疫球蛋白亚型相关（IgG亚型的风险为4.7％和3.5％；IgA亚型的风险为20.5％和14.0％）。Eythorsson等[11]利用大宗人群队列研究iStopMM中1 043例有骨髓样本的MGUS数据，建立了一个可以预测MGUS患者骨髓浆细胞是否≥10％的多变量预测模型。模型包括MGUS的单克隆类型，单克隆蛋白浓度，sFLC，总IgG、IgM和IgA浓度，预测模型的c统计量为0.85，在预测SMM或更严重情况的10％风险阈值下，敏感性为86％，特异性为67％，阳性预测值为32％，阴性预测值为96％。此模型可以在www.istopmm.com/riskmodel直接输入数值计算，但该研究目前仅有冰岛患者的数据，并未进行外部验证。考虑到骨髓穿刺对于M蛋白相关疾病的诊断和鉴别诊断非常重要，若不进行骨髓穿刺，有超过5％的漏诊率。因此，本共识推荐至少完善一次骨髓穿刺细胞形态学及流式细胞术检查以排除其他浆细胞或B淋巴细胞疾病。

在细胞遗传学异常方面，不同国家报道的发生率相差较大。北京大学人民医院一项纳入111例MGUS患者的研究中，10.7％的患者常规细胞遗传学检测异常，而荧光原位杂交（fluorescence in situ hybridization，FISH）使阳性率增加到18.9％，IgH重排最为常见[12]。另外一些报道显示，在非IgM MGUS中，有接近半数患者发现涉及免疫球蛋白重链（IGH）基因在14q32和5个伙伴染色体易位［11q13（最常见的是cyclin D1基因）、4p16.3（FGFR-3和MMSET基因）、6p21（cyclin D3基因）、16q23（C-MAF基因）和20q11（MAFB基因）］[13]–[14]。除了IGH易位外，在MGUS中也检测到了已知的MM特异性染色体异常，如RB1（13q14）缺失、1q扩增和超二倍体；但上述异常在MGUS中的发生频率比在MM中低[8],[15]。在MGUS患者中未检测到TP53或MYC的突变或缺失，表明上述事件可能发生在病程后期[16]。IgM MGUS中则常发现MYD88 L265P和CXCR4突变，分别占60％和9％[17]。

（3）影像学检查：在M蛋白浓度<15 g/L的患者骨骼检查中发现骨病变的概率非常低，不同影像学方法检测的敏感性不同。其中X线检测IgG型M蛋白患者骨病变的发生率为1.7％，IgA型为6.4％[9]。

全身电子计算机断层扫描（whole body computed tomography，WB CT）、全身磁共振成像（whole body magnetic resonance imaging，WB MRI）和全身正电子发射计算机体层扫描术（positron emission tomography，PET）/CT等方法在发现骨骼病灶和淋巴结肿大等方面均优于传统骨骼X线检查。IMWG的一项多中心分析比较了212例单克隆浆细胞疾病患者采用传统骨骼X线和WB CT的检查结果，发现25.5％的传统骨骼X线检查阴性患者的WB CT呈阳性[18]。一系列报道显示，WB MRI检测M蛋白患者发现局部病变的比例为3.5％～23.4％[19]–[21]。在M蛋白患者的检测中，全身PET/CT较传统X线检查更为敏感，可发现4.4％～9.5％患者存在X线检查未发现的病灶[22]。在某些特定患者中，当WB CT结果不明确而有理由怀疑MM时，WB MRI可能具有诊断价值。如老年性骨质疏松与骨髓瘤恶性浸润引起的骨质疏松可通过WB MRI鉴别。IgM型MGUS通常会发展为LPL/WM，较罕见发展为MM。对这些患者可应用全身PET/CT或WB CT检查。

（4）其他检查：在有临床表现时，需要完善相关检查排除其他浆细胞疾病：脑钠肽（brain natriuretic peptide，BNP）或氨基末端BNP前体（N-terminal pro b-type natriuretic peptide，NT-proBNP）、肌钙蛋白、血管内皮生长因子（vascular endothelial growth factor，VEGF）、肌电图、眼底镜检查、心脏彩超、胃肠镜、肾脏穿刺活检、淋巴结活检、皮肤活检等。

本共识推荐：

①所有患者均应接受血常规、血清肌酐（估算肾小球滤过率）、血清钙（计算校正钙）、血清乳酸脱氢酶、β_2_微球蛋白、血清白蛋白、血清免疫球蛋白定量、sFLC、血清蛋白电泳、血清免疫固定电泳、尿微量蛋白定量、24 h尿蛋白定量、24 h尿轻链定量、尿免疫固定电泳检测。

②所有怀疑MGUS的患者均建议完善至少一次骨髓检查：包括骨髓细胞学涂片、流式细胞术检测（包括浆细胞表型和B淋巴细胞表型）、骨髓活检+免疫组化。对于非IgM型MGUS，推荐对骨髓浆细胞分选后行FISH检测［检测探针至少包括13q−、t（4;14）、t（11;14）、t（14;16）、1q21扩增和1p32−］；对于IgM型MGUS，推荐检测MYD88 L265P和CXCR4。

③建议进行全身影像学检查，包括PET/CT、WB MRI或WB CT检查；若条件不允许，也可行常规骨骼X线检查，以排除MM（非IgM型）或LPL/WM（IgM型）。

④有相关临床表现时，建议完善以下检查排除其他浆细胞疾病：BNP或NT-proBNP、肌钙蛋白、VEGF、肌电图、眼底镜检查、心脏彩超、胃肠镜、肾脏穿刺活检、淋巴结活检、皮肤活检等。

4. 诊断标准：本共识诊断标准参照2014年IMWG诊断标准[6]和《中国多发性骨髓瘤诊治指南（2024年修订）》[7]的诊断标准。按照单克隆免疫球蛋白类型可分为非IgM型（多为IgG型或IgA型）、IgM型和轻链型，相应的诊断标准如下：

（1）非IgM型MGUS：满足以下所有条件：血清单克隆免疫球蛋白（非IgM型）<30 g/L；骨髓克隆性浆细胞<10％；无浆细胞异常增殖引起的终末器官功能损害，如高钙血症、肾功能不全、贫血、骨质破坏、淀粉样变等。

（2）IgM型MGUS：满足以下所有条件：血清单克隆IgM蛋白<30 g/L；骨髓淋巴样浆细胞<10％；无贫血、全身症状、高黏滞综合征、淋巴结肿大、肝脾肿大或其他淋巴细胞增殖性疾病导致的终末器官功能损害。

（3）轻链型MGUS：满足以下所有条件：sFLC比值异常（<0.26或>1.65）；受累轻链水平增加（在比值>1.65的患者中，κ游离轻链增加；在比值<0.26的患者中，λ游离轻链增加）；免疫固定电泳未发现重链克隆性表达；骨髓克隆性浆细胞<10％；尿单克隆轻链定量<500 mg/24 h；无浆细胞异常增殖引起的终末器官功能损害，如高钙血症、肾功能不全、贫血、骨质破坏、淀粉样变等。

5. 鉴别诊断：一旦检测出单克隆免疫球蛋白，除需与SMM或AMM鉴别外，还需要与其他M蛋白相关疾病进行鉴别，如AL、POEMS综合征或各种具有临床意义的单克隆免疫球蛋白血症（monoclonal gammopathy of clinical significance，MGCS）如MGRS、IgM相关性周围神经病变等。伴有不明原因骨质疏松的MGUS患者也应仔细检查，以确保骨质疏松不是由MM引起。此外，某些类型的非霍奇金淋巴瘤、伴或不伴浆细胞分化的B淋巴细胞增殖性疾病也可能出现M蛋白，应在鉴别诊断时予以考虑。

（1）MM：MGUS通常无症状，M蛋白水平<30 g/L，骨髓浆细胞浸润<10％；而MM则表现为骨质破坏、肾功能不全、贫血和高钙血症（CRAB），M蛋白水平常较高，骨髓异常浆细胞超过10％。需要注意的是，CRAB并非MM所特有，也可在其他疾病中出现，此时合并M蛋白血症需与MM鉴别。如合并糖尿病肾病、高血压肾病的患者往往会出现肾功能不全和贫血；合并骨多发血管瘤/脂肪瘤的患者可出现骨质破坏，这时需要鉴别这些器官损害是由原发病引起还是与M蛋白相关。

（2）AL：淀粉样蛋白沉积在组织中导致器官功能障碍，如心脏、肾脏或神经病变。AL的确诊需要组织活检证实淀粉样蛋白沉积。与MGUS相比，AL患者通常有显著的器官受累症状。

（3）POEMS综合征：POEMS综合征是一种罕见的与浆细胞异常相关的多系统疾病，特征包括多发性神经病、器官肿大、内分泌紊乱、M蛋白和皮肤改变。

（4）MGRS：MGRS患者有单克隆免疫球蛋白血症，并造成显著的肾脏受累，如出现蛋白尿、肾病综合征或肾功能不全。肾脏病理是诊断MGRS的金标准。有无肾脏损害可鉴别MGRS和MGUS。

（5）其他M蛋白相关疾病：包括但不限于LPL/WM、重链病、IgM相关性周围神经病变等。上述疾病通常有特定的临床表现、实验室检查特点和治疗需求。如LPL/WM患者有高黏滞综合征和淋巴结肿大等表现，M蛋白多为IgM型，骨髓流式细胞术可以检测出克隆性B淋巴细胞和（或）克隆性浆细胞等，MYD88 L265P突变常阳性。

6. 预后评估：MGUS进展为MM或LPL/WM的发生率约为每年1％。不同类型的MGUS进展为不同种类的疾病（表1），其中IgM型MGUS进展为LPL/WM的可能性远高于MM[6]。

**表1:** 不同类型意义未明的单克隆免疫球蛋白血症（MGUS）进展疾病种类和风险

MGUS类型	进展的疾病种类	进展风险
非IgM型MGUS（IgG、IgA）	多发性骨髓瘤、孤立性浆细胞瘤、免疫球蛋白相关的淀粉样变性（AL、AHL、AH）	每年1％
IgM型MGUS	淋巴浆细胞淋巴瘤/华氏巨球蛋白血症、B细胞非霍奇金淋巴瘤、免疫球蛋白相关的淀粉样变性（AL、AHL、AH）	每年1.5％
轻链型MGUS	轻链型多发性骨髓瘤、轻链型淀粉样变性	每年0.3％

**注** AL：轻链型淀粉样变性；AHL：轻重链淀粉样变性；AH：重链淀粉样变性

许多生物学特征具有预后预测价值，如M蛋白类型、sFLC比值异常、存在尿本周蛋白、检测到循环浆细胞、流式细胞术检测的异常浆细胞比例、细胞遗传学异常、M蛋白的快速进行性增加等均与MGUS的进展有关[9]。目前已经发表了多个预测MGUS进展的风险分层模型，其中常被引用的模型是梅奥诊所2005模型和西班牙2010年模型。

在梅奥诊所的风险分层模型中[23]，3个风险因素定义为sFLC比值异常（<0.26或>1.65）、血清M蛋白浓度高（>15 g/L）、非IgG亚型（IgA型或IgM型）。在初次就诊时，可以根据存在的风险因素数量对MGUS患者进行分层：没有风险因素为低危组，20年进展风险为2％；含有1个风险因素为低中危组，20年进展风险为10％；含有2个风险因素为中高危组，20年进展风险为18％；含有3个风险因素为高危组，20年进展风险为27％。虽然梅奥模型发表时间较早，目前有其他模型可能优于该模型，但该模型是目前国际上应用最广泛、最简便且无需骨髓检查结果的预测模型。

西班牙PETHEMA研究小组2007年发布了一个MGUS预后模型，发现DNA非整倍体和骨髓异常/正常浆细胞比值≥95％是预测MGUS进展的独立变量。0个危险因素患者5年进展率为4％，1个危险因素患者5年进展率为46％，2个危险因素患者5年进展率为72％。这个模型需要进行骨髓检查[24]。同一研究小组2010年在原模型的基础上进一步探索了疾病的演变模型对预后的预测作用，将进展性MGUS定义为随访第3年M蛋白间隔两个月连续性检测增加≥10％，结合原来的异常/正常浆细胞比值≥95％的预后因素建立了一个新的预后模型，提供了更为全面的进展风险评估。无危险因素患者7年内累积进展率为2％，1个危险因素为16％，2个危险因素为72％[25]。该模型目前在国际上的应用也较为广泛且使用简便。

2019年，Landgren等[26]基于美国国家癌症所（National Cancer Institute，NCI）前瞻性数据开发了一个新的预后评分模型。该模型的危险因素包括IgA亚型、单克隆免疫球蛋白浓度≥15 g/L、sFLC比值<0.1或>10及免疫抑制程度（1种或2种未受累的免疫球蛋白水平低于正常值）。具体分组如下：低危组：0～1分；中危组：2分；高危组：≥3分。且该模型还推荐对MGUS患者的预后进行动态分析。但该模型缺乏外部验证，目前尚未广泛应用。

2023年，Cowan等[27]开发了一个新的根据动态生物标志物预测MGUS或SMM进展为MM的多变量预测模型——PANGEA模型。这项回顾性多队列研究共纳入6 441例患者。该模型通过多变量Cox回归分析找到了6个重要的进展预测因子，包括sFLC比值、单克隆免疫球蛋白浓度、年龄、肌酐浓度、血红蛋白下降水平和骨髓浆细胞百分比，并开发了有骨髓数据和无骨髓数据两种版本的PANGEA模型，通过计算患者进展为MM的风险评分，反映该患者在特定时间内（1年、2年、5年、10年和25年）进展为MM的概率。此模型可以在https://www.pangeamodels.org/计算，但模型目前还未得到外部验证，且计算复杂，需要使用在线工具进行计算，使用不便利。

考虑到2019年和2023年两个预后模型建立时间较短，验证不充分，目前尚不推荐临床使用。

本共识推荐：使用Mayo 2005模型和西班牙2010年模型进行预后评估。

7. 患者管理：

（1）是否治疗：虽然目前有关于高危MGUS提早治疗是否可以治愈这类疾病的临床研究，但鉴于目前临床数据和观察的时间有限，不推荐对MGUS患者进行除临床研究外的其他治疗。

本共识推荐：对于所有MGUS，除非临床研究，不推荐启动治疗。强烈建议对所有确诊患者进行规范化管理和随诊。

（2）随诊：MGUS患者管理的目的是早期发现进展为MM或LPL/WM的患者，及时启动治疗以减少严重并发症并延长生存时间。目前没有关于MGUS患者定期监测最佳频率的前瞻性数据发表。目前关于MGUS最大宗的随访数据来自SEER数据库的一项回顾性分析[28]，在17 457例MGUS患者中，1 037例（6％）进行了定期监测，定期监测组较非定期监测组在确诊MM时有较少的主要并发症（*HR*＝0.68，95％ *CI* 0.57～0.80）和较长的总生存（OS）期（中位OS期：23个月对19个月，*P*<0.001；*HR*＝0.87，95％ *CI* 0.80～0.95）。而梅奥诊所进行的一项回顾性分析表明，对于有MGUS病史的症状性骨髓瘤患者（116例），至少每3年进行1次定期监测并未减少住院治疗比例或骨髓瘤相关并发症[29]。

对于所有新诊断为MGUS的患者推荐在诊断后6个月进行血液检查（血常规、肌酐、血清钙、M蛋白和sFLC水平），此后低危患者可每1～2年复查1次，中高危患者每年复查1次，或出现提示浆细胞恶性肿瘤的症状时随时复诊。

除M蛋白水平升高外，如果出现以下任何无法解释的症状、体征或实验室检查异常，均应考虑是否进展：进行性的无法解释的疼痛、骨折、贫血、红细胞沉降率升高、非贫血引起的呼吸急促、肝肿大、腹泻、高钙血症、高黏滞综合征、假性肠梗阻、巨舌症、肾病综合征、神经病变（自主神经、感觉神经或运动神经）、皮肤紫癜和肾功能不全等。若出现上述情况，需要尽快完善MM、AL、LPL/WM等疾病的相应检查。

本共识推荐：

①随访时间间隔：对于所有新诊断为MGUS的患者应在诊断后6个月进行血液学复查，此后低危患者可每1～2年复查1次，中高危患者每年复查1次，或出现提示浆细胞恶性肿瘤的症状时随时复诊。在常规复诊期间，若M蛋白水平升高5 g/L及以上和（或）受累sFLC水平增加10 mg/dl及以上，则应调整为每1～3个月复诊1次。

②随访内容：复诊的检测内容至少包括M蛋白定量、血常规、血钙、血清肌酐和sFLC。若临床怀疑疾病进展，需复查骨髓和影像学检查。

8. 筛查：虽然MGUS没有临床症状，但有报道显示MGUS已与100多种临床表现有一定的相关性，包括血栓形成、感染、自身免疫病、骨折、神经病变，且有报道显示MGUS的死亡率高于普通人群。因此目前关于是否需要对所有人群进行MGUS筛查有较大争议。但需要明确的是，大多数MGUS患者是因其他疾病去医院就诊的过程中诊断MGUS，因此，上述疾病与MGUS之间的相关性可能被高估。目前唯一一个关于大宗人群MGUS筛查的研究——iStopMM研究，是一项基于所有人群的筛查研究和随访策略的随机对照试验，评估了MGUS筛查的潜在益处和危害。早期结果表明，该研究强化随访组患者LPL/WM、SMM和AMM的检出率更高，主动筛查出的MGUS患者合并症更少[30]。该研究结果显示，通过筛查可能提高这些恶性肿瘤的检出率，但还需要进行更长期的随访以确定检出率的提高能否转化为患者更好的预后，并评估筛查对患者造成的心理负担。考虑到目前没有证据支持对所有人群进行MGUS筛查可以转化为患者更好的预后，本共识不推荐对所有人群进行常规筛查。但考虑到主动筛查会降低MM晚期诊断的风险，从而降低MM相关事件带来的不良预后或死亡风险，本共识推荐可以对高危人群进行MGUS筛查，高危人群指其一级亲属患有浆细胞疾病（如MGUS、SMM、AMM）、LPL/WM或其他血液肿瘤，或有2个及以上家庭成员患血液系统肿瘤。

本共识推荐：由于目前没有证据支持早期大规模人群筛查可以转化为患者更好的预后，所以仅对高危人群进行M蛋白筛查。

二、SMM

1. 流行病学：SMM的流行病学尚不明确，目前关于SMM的流行病学调查几乎均来自于欧美国家。瑞典骨髓瘤登记系统的数据表明，SMM的发病率为0.4/100 000每年。iStopMM研究筛查了冰岛所有40岁以上的成年人，包括75 422例患者，SMM的总体患病率为0.53％，男性多见，中位年龄为70岁[4],[30]–[31]。SMM的发病率随年龄增长而升高，70岁以上为1.08％，80岁以上为1.59％。

2. 临床表现：基于2014 IMWG[6]和《中国多发性骨髓瘤诊治指南（2024年修订）》[7]中关于SMM的诊断标准，SMM除了有达到诊断MM的M蛋白水平和克隆性浆细胞以外，没有浆细胞异常增殖引起的终末器官功能损害，如骨髓瘤定义事件SLIM-CRAB；也没有其他M蛋白直接或间接引起的器官损害，如AL、M蛋白相关脏器损害（肾脏、心脏、血管、神经、眼部、肌肉、皮肤等）。若临床出现上述症状，必须排除与M蛋白相关才能诊断SMM。

3. 检验和检查：

（1）血液/尿液检查：诊断SMM时，不仅要排除有症状的MM、LPL/WM，还要考虑其他与M蛋白相关的疾病，如AL、POEMS综合征、MGRS、IgM相关性周围神经病变等，因此，所有患者均应接受血常规、血清肌酐（估算肾小球滤过率）、血清钙（计算校正钙）、血清乳酸脱氢酶、β_2_微球蛋白、血清白蛋白、血清免疫球蛋白定量、sFLC、血清蛋白电泳、血清免疫固定电泳、尿微量蛋白定量、24 h尿蛋白定量、24 h尿轻链定量、尿免疫固定电泳检测。

（2）骨髓细胞学和流式细胞术检查：所有怀疑SMM的患者均需要完善骨髓检查，包括骨髓细胞学涂片、流式细胞术检测（包括浆细胞表型和B淋巴细胞表型）、骨髓活检+免疫组化和骨髓浆细胞FISH检测。推荐对骨髓浆细胞分选后行FISH检测［检测探针至少包括13q−、t（4;14）、t（11;14）、t（14;16）、1q21扩增、1p32−和17p−］。

（3）影像学检查：骨骼X线检查既往是评估MM患者骨病的标准方法。但这种技术敏感性较低，与目前先进的成像技术相比有明显的局限性。全身低剂量CT在脊柱、骨盆、颅骨和肋骨等区域优于骨骼X线检查[32]。WB MRI在可疑溶骨或骨质疏松部位是否存在骨髓浸润的诊断上具有重要意义[33]。一项系统性综述对18项比较^18^F-FDG PET/CT与全身X线或MRI或两者的研究进行了评估，确认MRI是评估脊柱弥漫性骨髓受累的金标准技术，而^18^F-FDG PET/CT在检测骨病变方面较全身X线更敏感[34]。

本共识推荐：

①所有患者均应接受血常规、血清肌酐（估算肾小球滤过率）、血清钙（计算校正钙）、血清乳酸脱氢酶、β_2_微球蛋白、血清白蛋白、血清免疫球蛋白定量、sFLC、血清蛋白电泳、血清免疫固定电泳、尿微量蛋白定量、24 h尿蛋白定量、24 h尿轻链定量、尿免疫固定电泳检测。

②所有怀疑SMM的患者均需完善骨髓检查：包括骨髓细胞学涂片、流式细胞术检测（抗体至少包括B细胞和浆细胞检测）、骨髓活检+免疫组化和骨髓浆细胞FISH检测［检测探针至少包括13q−、t（4;14）、t（11;14）、t（14;16）、17p−、1q21扩增和1p32−］。

③建议完善横断面影像学检查，推荐应用PET/CT、WB MRI或WB CT评估骨病。若不可及，可考虑骨骼X线检查。骨髓影像学检查为诊断AMM的必备检查，若存在骨病，则不能诊断SMM。

④有相关临床表现时，建议完善以下检查排除其他浆细胞疾病：BNP或NT-proBNP、肌钙蛋白、VEGF、肌电图、眼底镜检查、心脏彩超、胃肠镜、肾脏穿刺活检、淋巴结活检、皮肤活检等。

4. 诊断标准：本共识诊断标准参照2014 IMWG诊断标准[6]和《中国多发性骨髓瘤诊治指南（2024年修订）》的诊断标准[7]。

（1）血清M蛋白≥30 g/L或24 h尿单克隆轻链≥0.5 g和（或）骨髓单克隆浆细胞比例10％～59％；

（2）无骨髓瘤定义事件（SLiM-CRAB）或淀粉样变。

5. 预后评估：SMM是异质性较大的疾病，有些患者可能很长时间维持在SMM阶段，而另一些患者可能会迅速进展为AMM。SMM进展到AMM的中位时间约为5年。在确诊后的前5年，进展到AMM的风险约为每年10％；接下来的5年，进展风险降低至每年3％；之后，进展风险进一步降低至每年1％。

对SMM患者进行准确的预后评估对于决定这些患者的监测频率和治疗时机十分重要。多种因素可能会影响SMM患者的预后，包括骨髓中克隆性浆细胞比例、M蛋白的类型和水平、尿轻链水平、sFLC比值、免疫抑制、循环浆细胞比例和细胞遗传学异常等。目前有多种预后评估模型，包括PETHEMA模型[24]、Mayo-2008模型[35]、Mayo-2018模型[36]和IMWG-2020模型[37]等，2014年IMWG诊断标准更改，前两者现已不适用于SMM的危险评估，目前Mayo-2018模型应用最广泛。这个模型根据417例SMM患者的回顾性研究得出，根据3个指标对SMM进行风险分层，简称为20/20/20，包括克隆浆细胞>20％，M蛋白>20 g/L和sFLC比值>20（详见表2）。

**表2:** 冒烟型多发性骨髓瘤的推荐预后模型

模型	危险因素	危险分组	危险因素个数/分数^a^	例数	2年进展率（％）	中位无进展生存期（月）
Mayo-2018	克隆浆细胞>20％、M蛋白>20 g/L、sFLC比值>20	低危组	0	143	9.7	110
		中危组	1	121	26.3	68
		高危组	≥2	153	47.4	29
IMWG-2020	克隆浆细胞>20％、M蛋白>20 g/L、sFLC比值>20、高危细胞遗传学［t（4;14）、t（14;16）、1q扩增、del（13q）］	低危组^b^	0	225	6.0	–
		低-中危组^b^	1	224	22.8	–
		中危组^b^	2	177	45.5	–
		高危组^b^	3～4	63	63.1	–
		低危组^c^	0～4	241	3.8	–
		低-中危组^c^	5～8	264	26.2	–
		中危组^c^	9～12	133	51.1	–
		高危组^c^	>12	51	72.5	–

**注** ^a^分数：sFLC比值 0～10：0分，10～25：2分，25～40：3分，>40：5分；M蛋白（g/L）0～15：0分，15～30：3分，>30：4分；骨髓浆细胞比例（％）0～15：0分，15～20：2分，20～30：3分，30～40：5分，>40：6分；高危细胞遗传学：无：0分，有：2分。^b^按照危险因素个数分组。^c^按照危险因素分数分组。–：无数据

IMWG-2020模型是一项更大规模的多中心回顾性研究，研究对象为1 996例根据2014年IMWG标准诊断的SMM患者，进一步实现了Mayo-2018模型的价值。在此基础上，在689例具有完整数据集的患者中进行了额外分析，预后因素增加了高危细胞遗传学异常，汇总数据确定了四个危险组，根据危险因素个数分为低危组、低-中危组、中危组和高危组，随后进一步根据每个危险因素值的范围赋分计算后进行分组（详见表2）。考虑到计分方法实际应用便利性较差，推荐选用IMWG-2020模型中按照危险因素个数分组进行预后评估。

此外，2023年发布的PANGEA模型[27]（https://www.pangeamodels.org/）纳入6个进展预测因素，包括sFLC比值、单克隆免疫球蛋白浓度、年龄、肌酐浓度、血红蛋白下降水平和骨髓浆细胞百分比。在SMM患者中，PANGEA模型预测进展为MM的准确性较IMWG 20/20/20模型更高。但这个模型目前还未得到外部验证，且计算复杂，需要使用在线工具进行计算。

综合应用广泛、使用便利性及临床接受度，本共识推荐使用Mayo-2018模型和IMWG-2020模型中按照危险因素个数分组对SMM患者进行预后评估。

本共识推荐：使用Mayo-2018模型和IMWG-2020模型中按照危险因素个数分组对SMM患者进行预后评估。

6. 患者管理：

（1）是否治疗：一直以来，SMM的传统管理方法是密切观察，待疾病进展为AMM时才启动治疗。随着对疾病生物学理解的深入、危险分层的改进及各种疗效更好、不良反应更少的新治疗方案的引入，研究者们开始探讨SMM患者早期干预的意义。

目前关于高危SMM提前治疗的多个临床研究正在进行中，主要可分为两种治疗目标：一种是以控制疾病为目标的低强度治疗，另一种是以治愈疾病为目标的强化治疗。

低强度治疗最具代表性的两个Ⅲ期临床研究分别为QuiRedex[38]–[40]和ECOG E3A06[41]。QuiRedex研究纳入119例高危SMM患者，随机分为来那度胺、地塞米松组（Rd）和观察组，中位随访时间为12.5年，观察组的中位疾病进展时间为2.1年，而Rd组为9.5年（*HR*＝0.28，95％ *CI* 0.18～0.44，*P*<0.001）。观察组中位OS期为8.5年，而Rd组未达到（*HR*＝0.57，95％ *CI* 0.34～0.95，*P*＝0.032）。尽管这项研究显示了早期干预高危SMM的整体生存获益，但这项试验招募患者的时间为2007年至2010年，是在更新IMWG 2014年诊断标准之前，且未使用敏感的影像学检查，该研究实际入组了部分现在被定义为AMM的患者。因此，该临床研究的结果至今仅能提供参考。另一个重要的Ⅲ期试验是ECOG E3A06[41]。该研究入组时间为2013年至2017年，共纳入182例中高危SMM患者，随机分为单药来那度胺治疗组和观察组。来那度胺组的总体有效率（ORR）为50％。1年、2年和3年无进展生存（PFS）率分别为98％、93％和91％，而观察组分别为89％、76％和66％。来那度胺组血液学和非血液学3级不良事件更多（35.2％）。亚组分析中，在高危SMM患者中观察到PFS获益，但在低中危患者中无明显获益。由于本研究入组时间较早，根据后来的Mayo 2018模型进行分层，本研究入组患者仅56例（30.8％）为真正的高危SMM，且其中仅不到半数（25例）入组研究组，存在统计效能不足的问题，且当时部分入组患者按目前标准而言已经是MM而非SMM。

强化治疗的临床研究包括GEM-CESAR[42]、ASCENT[43]临床研究等。GEM-CESAR是一个Ⅱ期单臂临床研究，纳入90例年龄<70岁、适合移植的高危SMM患者。患者接受6个周期的卡非佐米、来那度胺和地塞米松（KRd）治疗，序贯自体造血干细胞移植，随后接受2个疗程KRd方案巩固和2年来那度胺维持治疗。维持治疗结束时，47例患者MRD阴性，其中23例患者在治疗后2年MRD持续阴性。ASCENT研究入组了87例Mayo-2018模型定义的高危SMM患者，采用固定2年24个周期的达雷妥尤单抗联合卡非佐米、来那度胺和地塞米松治疗。结果显示84％的患者达到MRD阴性，3年PFS率为89.9％。15％的患者发生3级或以上血液学不良反应，51％的患者发生非血液学不良反应。

基于上述临床研究结果，考虑部分临床研究入组人群与目前诊断标准存在差异，对结果判断造成一定的困扰，且大多数研究随访时间较短，结合经济学和二重肿瘤等不良反应的考虑，本共识推荐，除非进入临床研究，对于高危SMM也不推荐提前干预治疗。对于所有危险分层的SMM，推荐定期监测和随访。

本共识推荐：对于高危SMM，除非进入临床研究，否则不推荐提前干预治疗。对于所有危险分层的SMM，推荐定期监测和随访。

（2）随诊：对于所有SMM患者均需要定期进行复诊，复诊内容包括实验室检查和影像学检查。实验室检查内容包括血常规、血清肌酐（估算肾小球滤过率）、血清钙（计算校正钙）、血清白蛋白、血清免疫球蛋白定量、sFLC、血清蛋白电泳（包括M蛋白含量）、血清免疫固定电泳检测、尿微量蛋白定量、24 h尿蛋白定量、24 h尿轻链定量、尿免疫固定电泳。若临床怀疑进展，需要复查骨髓穿刺和全身影像学检查。影像学检查推荐尽量使用与确诊时相同的检查技术。

本共识推荐：

①随访时间间隔：对于低、中危SMM患者，建议在确诊后第1～2年每3个月进行1次实验室检查，后续如果病情稳定可考虑延长至每4～6个月1次；对于高危患者，推荐确诊后的至少前5年内每3个月进行1次实验室检查，并考虑每年重复影像学检查。当临床怀疑疾病进展时应随时复查实验室检查、骨髓穿刺和全身影像学检查。

②随诊内容：实验室检查复查内容包括血常规、血清肌酐（估算肾小球滤过率）、血清钙（计算校正钙）、血清白蛋白、血清免疫球蛋白定量、sFLC、血清蛋白电泳（包括M蛋白含量）、血清免疫固定电泳检测、尿微量蛋白定量、24 h尿蛋白定量、24 h尿轻链定量、尿免疫固定电泳。骨髓检查：骨髓涂片、流式细胞术、FISH、活检。影像学检查：PET/CT、WB MRI、WB CT。
